# MicroRNA Biogenesis Is Required for Mouse Primordial Germ Cell Development and Spermatogenesis

**DOI:** 10.1371/journal.pone.0001738

**Published:** 2008-03-05

**Authors:** Katsuhiko Hayashi, Susana M. Chuva de Sousa Lopes, Masahiro Kaneda, Fuchou Tang, Petra Hajkova, Kaiqin Lao, Donal O'Carroll, Partha P. Das, Alexander Tarakhovsky, Eric A. Miska, M. Azim Surani

**Affiliations:** 1 Wellcome Trust/Cancer Research United Kingdom Gurdon Institute, University of Cambridge, Cambridge, United Kingdom; 2 Advanced Research Technology, Applied Biosystems, Foster City, California, United States of America; 3 Laboratory of Lymphocyte Signaling, The Rockefeller University, New York, New York, United States of America; Institut Curie, France

## Abstract

**Background:**

MicroRNAs (miRNAs) are critical regulators of transcriptional and post-transcriptional gene silencing, which are involved in multiple developmental processes in many organisms. Apart from miRNAs, mouse germ cells express another type of small RNA, piwi-interacting RNAs (piRNAs). Although it has been clear that piRNAs play a role in repression of retrotransposons during spermatogenesis, the function of miRNA in mouse germ cells has been unclear.

**Methodology/Principal Findings:**

In this study, we first revealed the expression pattern of miRNAs by using a real-time PCR-based 220-plex miRNA expression profiling method. During development of germ cells, miR-17-92 cluster, which is thought to promote cell cycling, and the ES cell-specific cluster encoding miR-290 to -295 (miR-290-295 cluster) were highly expressed in primordial germ cells (PGCs) and spermatogonia. A set of miRNAs was developmentally regulated. We next analysed function of miRNA biogenesis in germ cell development by using conditional *Dicer*-knockout mice in which *Dicer* gene was deleted specifically in the germ cells. *Dicer*-deleted PGCs and spermatogonia exhibited poor proliferation. Retrotransposon activity was unexpectedly suppressed in *Dicer*-deleted PGCs, but not affected in the spermatogonia. In *Dicer*-deleted testis, spermatogenesis was retarded at an early stage when proliferation and/or early differentiation. Additionally, we analysed spermatogenesis in conditional *Argonaute2-*deficient mice. In contrast to *Dicer*-deficient testis, spermatogenesis in *Argonaute2*-deficient testis was indistinguishable from that in wild type.

**Conclusion/Significance:**

These results illustrate that miRNAs are important for the proliferation of PGCs and spermatogonia, but dispensable for the repression of retrotransposons in developing germ cells. Consistently, miRNAs promoting cell cycling are highly expressed in PGCs and spermatogonia. Furthermore, based on normal spermatogenesis in *Argonaute2*-deficient testis, the critical function of Dicer in spermatogenesis is independent of *Argonaute2*.

## Introduction

MicroRNAs (miRNA) ranging between 18–23 bp are critical regulators of transcriptional and post-transcriptional gene silencing in many organisms. Expression of miRNA is subject to tight temporal and spatial regulation [1], [2], and they exhibit specific functions during development and homeostasis. Generation of miRNAs is a multi-step process in which Dicer, the RNaseIII-containing enzyme, catalyses precursors of miRNA to form mature miRNAs [3], [4]. Mature miRNA is incorporated into the effector RNA-induced silencing complex (RISC) composed of Argonaute (Ago) proteins, which have RNaseIII activity [5]. In the mouse, only a single Dicer gene has so far been identified, and its disruption leads to defects in miRNA generation as well as in RNA interference-mediated gene silencing [6], [7]. Dicer-mediated miRNA biogenesis is required for multiple developmental processes, and not just in the initial differentiation of the early embryo [8] and embryonic stem (ES) cells [6], [7]. MiRNAs have a role in limb development [9], lung epithelial morphogenesis [10], embryonic angiogenesis [11], hair follicle formation [12], [13] and T-cell proliferation [14]. Furthermore, miRNAs are also suggested to play a role in repression of retrotransposons in mice, based on the evidence that retrotransposons were up-regulated in the *Dicer*-deficient ES cells [6] and Dicer-hypomorphic preimplantation embryo [15]. However, the functions of miRNAs in germ cell development remain unclear.

Apart from miRNAs, another type of small RNAs has been recently isolated from germ cells. These are Piwi-interacting RNA (piRNA) [16]–[18], or germline small RNA (gsRNA) [19] of approximately 26–30 bp, which are specifically expressed in the testis. PiRNAs interact with mouse piwi-family proteins, such as Miwi, Miwi2 and Mili. These piwi-family proteins play an essential role in spermatogenesis. Spermatogenesis was arrested at the spermatid stage in *Miwi*-deficient mice [20], and as spermatocytes in *Miwi2*- or *Mili*-deficient mice [21], [22]. Furthermore, expression of retrotransposons was elevated in Miwi2- and Mili-deficient testis, coupled with a loss of DNA methylation at these loci [21], [23]. These observations indicate that piRNAs control repression of retrotransposons through DNA methylation, which is essential for spermatogenesis [24]. During spermatogenesis, piwi-family proteins are associated with a germ cell-specific perinuclear granule called nuage or chromatoid body [25], [26]. Similarly, Dicer and other Argonaute proteins are also localised in the chromatoid body [26], indicating that these pathways may interact.

In this study, we reveal the expression pattern and functional requirements of miRNA in PGC development and early spermatogenesis. During development of germ cells, miR-17-92 and miR-290-295 clusters were highly expressed. Additionally, a set of miRNAs was developmentally regulated. *Dicer*-deleted PGCs exhibited poor proliferation. Furthermore, retrotransposons activity was unexpectedly suppressed in *Dicer*-deleted PGCs, but not affected in th spermatogonia of neonatal animals. Furthermore, spermatogenesis was retarded in the *Dicer*-deficient testis at an early stage when proliferation and/or early differentiation of these cells commence before their entry into meiosis.

## Results

### Enrichment of oncogenic and ES cell-specific miRNAs in PGCs

To identify miRNAs that are expressed during PGC development, we performed real-time PCR analyses using primers containing stem-loop structure by which miRNAs can be representatively amplified from a small number of cells, and indeed, from single cells [27], [28]. In this study, we isolated 10 PGCs following FACS-sorting of Oct4-GFP positive germ cells from embryos at E9.5-E11.5 [29]. PGCs from female and male embryos were collected separately from E12.5 and E13.5 embryos. The PGCs were then subjected to the real-time PCR to assess expression of 214 known miRNAs listed in Figure S1.

**Figure 1:**
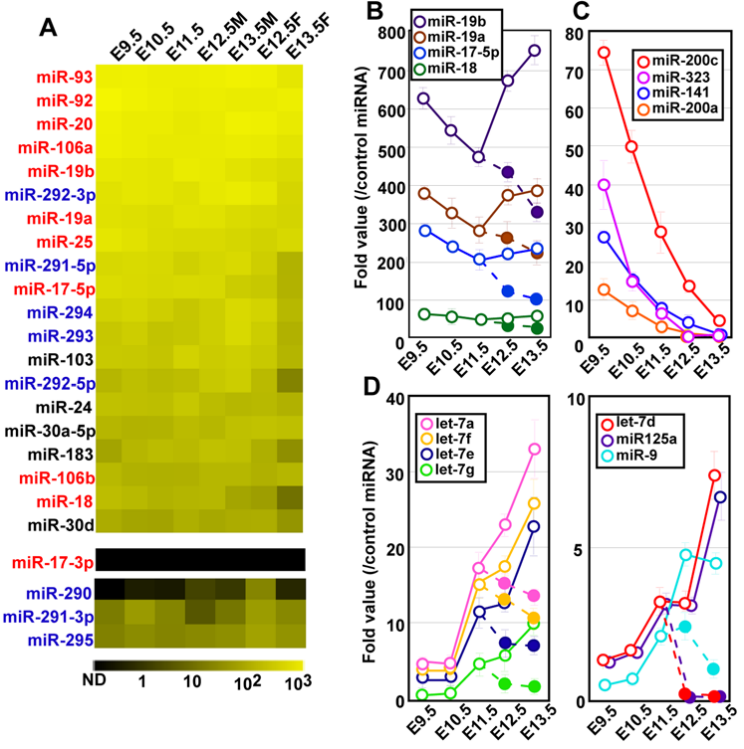
The miRNA expression during PGC development. (A) High expression of miR-17-92 and miR-290-295 clusters. The miRNA levels detected by real-time PCR are represented as a heat map. The intensity of yellow scale corresponds to the level of miRNA expression; black indicates that expression was undetectable. MiRNAs encoded in miR-17-92 and miR-290-295 clusters are marked with red and blue, respectively. (B-D) Alteration of miRNA expression during PGC development. The relative values of miRNA expression in male (solid line and open cycles) and female (broken line and closed circles) after E12.5 are shown. Until E11.5, the sex of PGCs was not determined. Relative values were calculated by dividing the raw value of the individual miRNA by that of the recombinant miR-16 as its levels were pre-determined in the samples.

Amongst the miRNAs detected in the samples, miRNAs belonging to miR-17-92 cluster was highly expressed in PGCs. All miRNAs, in miR-17-92 cluster except for miR-17-3p were amongst the top twenty highly expressed miRNAs (Figure 1A and Figure S2), but miR-17-3p was undetectable (Figure 1A). The expression levels of miRNAs were maintained throughout development of PGCs, although the expression of some miRNAs (miR-17-5p, -18, -19a and -19b) decreased gradually in female PGCs after E12.5 when they prepare to enter into meiotic prophase. (Figure 1B). Since miR-17-92 cluster promotes cell survival and proliferation in mammalian cancer cells [30], [31], it is possible that miR-17-92 cluster may play a role in promoting the survival and/or the proliferation of PGCs.

**Figure 2:**
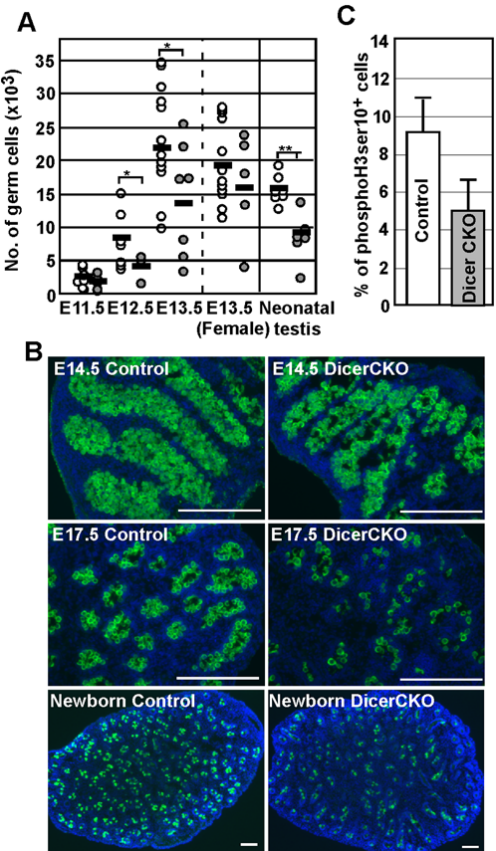
Defective proliferation of DicerCKO PGCs. (A) The number of PGCs in E11.5∼E13.5 gonads and in neonatal testes. A single dot shows the number of PGCs or germ cells in a pair of gonads or testes (open circles; Dicer^F/+^ TNAP-Cre, closed circles; DicerCKO). Black bar shows the mean. (B) Immunostaining for Mvh (green) in fetal and neonatal testis. The images are merged with DAPI (blue). Scale bar: 250 µm. (C) The reduced percentage of PGCs at M-phase in E12.5 DicerCKO male gonads. The percentages of PGCs immunostained for phosphorylated Ser10 of histone H3 are shown. Error bars represent standard deviation determined from three gonads.

In addition to the miR-17-92 cluster, we detected high expression of the ES cell-specific cluster, miR-290-295 cluster, in PGCs (Figure 1A and Figure S2). Expression levels of these miRNAs were maintained in both male and female germ cells at E12.5 and E13.5, except miR-291-5p and miR-292-3p both of which were slightly down-regulated in E13.5 female PGCs (Figure 1A). As with the expression of some pluripotent-specific genes such as Oct3/4, Nanog and Stella in PGCs, our results revealed that the ES cell-specific miRNAs were also expressed in PGCs.

### Developmental regulation of miRNAs in PGCs

In contrast to the constitutively high expression of miR-17-92 and miR-290-295 clusters, expression of some miRNAs was found to depend on the developmental stage of PGCs. Expression of 4 miRNAs (miR-141, -200a, -200c and -323) decreased gradually in developing PGCs (Figure 1C). The reduction in their expression was indistinguishable between male and female germ cells (Figure S2). Interestingly, three (miR-141, -200a and -200c) of these miRNAs belong to miR-200 superfamily, which has a similar seed sequence, which is thought to be important for the specificity of miRNA:mRNA recognition based on 3'UTR conservation [32], suggesting that these miRNA play a synergistic role during PGC development.

By contrast, expression levels of three miRNAs belonging to let-7 miRNA family (let-7a, d, e, f and g), as well as miR-125a and miR-9 increased in male PGCs but not in female PGCs (Figure 1D). These let-7 miRNAs share similar seed sequences, also suggesting their synergistic role in developing PGCs. In contrast to miR-17-92 cluster, the function of let-7 family is predicted as tumor suppressors through suppression of Ras/MAPK pathway [33], [34]. Furthermore, the expression of let-7 miRNAs is higher in differentiated cells compared to tumor cells [34]. These results indicate that these let-7 members may contribute to differentiation of male PGCs.

### Defective proliferation of Dicer-deleted PGCs

To clarify the function of miRNA in germ cell development, we generated mice lacking Dicer specifically in PGCs (DicerCKO) by crossing *Dicer*-floxed mice [13] with TNAP-Cre mice which express Cre preferentially in PGCs from E10 onwards [35]. As reported previously that Cre-mediated recombination of loxP sequence was observed in around 50% of the PGCs in E13.5 TNAP-Cre embryos [35], PCR analysis of genomic DNA from E13.5 PGCs showed that around 50% of the PGCs had their *dicer* allele deleted (Figure S3). Consistently, real-time PCR showed that the levels of *Dicer* expression were halved in DicerCKO E13.5 PGCs (Figure S3).

First, the number of PGCs in the DicerCKO embryos from E11.5 to E13.5 was assessed by FACS analysis using DicerCKO harboring the *Oct4ΔPE-GFP* reporter gene. In DicerCKO male fetal gonads at E12.5 and E13.5, the number of germ cells was significantly reduced (Figure 2A), whereas the number at E11.5 was comparable to the controls. The reduction in the number of PGCs was partially recovered in DicerCKO female fetal gonads at E13.5 (Figure 2A). In neonatal testis of DicerCKO, the number of neonatal spermatogonia was also reduced to almost half those detected in controls (Figure 2A and B). Note that the development of the seminiferous tubules in DicerCKO males is indistinguishable, compared to the controls (Figure 2B), suggesting that the reduction in the numbers of germ cells is due to an intrinsic defect in these cells.

The reduction in the numbers of PGCs in DicerCKO embryos might be caused either by cell death or by growth arrest of *Dicer*-deleted PGCs. To distinguish between these two possibilities, we assessed the number of apoptotic cells in the gonads. However, the number of apoptotic cells was not elevated in DicerCKO PGCs at E13.5 (Figure S4). Next we examined the proliferation rate of DicerCKO PGCs by immunostaining for phosphorylated histone H3 ser10, a marker of M phase. The immunostaining analysis revealed that the proliferation of DicerCKO PGCs at E12.5 was attenuated. On average, we found that 4.9% of PGCs in DicerCKO embryos were in M phase, whereas 9.0% of PGCs in control embryos were in M phase (Figure 2C). These results demonstrated that it is the proliferation, but not the survival, of PGCs which was impaired in DicerCKO gonads.

### Repression of IAP and LINE-1 expression in DicerCKO PGCs

**Figure 3:**
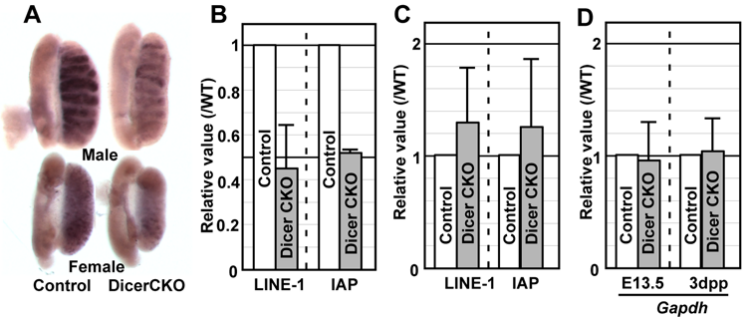
Lower expression of IAP and LINE-1 in DicerCKO PGCs. (A) In situ hybridization analysis of LINE-1 expression in E13.5 gonads. The signal was presumably detected in both male (upper) and female (bottom) germ cells. (B–C) Real-time PCR analysis of IAP and LINE-1 expression in E13.5 PGCs (B) and in neonatal spermatogonia (C). Shown are relative values of transcript levels of LINE-1 or IAP in DicerCKO, compared to those in littermate controls. (D) Real-time PCR analysis of *Gapdh* expression in E13.5 PGCs (E13.5) and neonatal spermatogonia (3dpp) in DicerCKO, compared to those in littermate controls. Shown are relative values of transcript levels of *Gapdh* in DicerCKO, compared to those in littermate controls. Same number of sorted PGCs from control and mutant embryos (3000 PGCs) were subjected to cDNA synthesis, followed by Q-PCR analysis. The value for *Gapdh* transcripts was calculated by using a reference graph generated from serially diluted DNA samples. The mean values in DicerCKO cells were calculated from three independent experiments.

We next addressed the function of Dicer in the regulation of retrotransposons during germ cell development. First, we assessed the expression of long interspersed nuclear element-1 (LINE-1), the most abundant family of non-LTR retrotransposons in the mammalian genome. Unexpectedly, *in situ* hybridization analysis revealed that LINE-1 repression was enhanced in E13.5 DicerCKO PGCs of both male and female gonads, compared to those in the controls (Figure 3A). This enhancement was confirmed by real-time PCR analysis using FACS-sorted PGCs. The levels of LINE-1 transcripts in DicerCKO PGCs were almost half of those detected in control PGCs (Figure 3B). In addition to LINE-1, the expression levels of the intracisternal A-particle (IAP) retrotransposable element was also halved in DicerCKO PGCs, compared to those in the control PGCs (Figure 3B). The DNA methylation levels, by which activation of retrotransposon are suppressed [36]–[39], was however comparable between DicerCKO and control PGCs at E13.5 (Figure S5). In addition to PGCs, we tested expression of retrotransposons in 3 days *post partum* (dpp) spermatogonia. The expression levels of the retrotransposons were comparable between DicerCKO and littermate controls (Figure 3C). In both E13.5 PGCs and 3dpp sprmatogonia, expression levels of *Gapdh* were also comparable between DicerCKO and controls (Figure3D), demonstrating that results of retrotransposon expressions were not due to alteration in the amount of *Gapdh* transcript. Although it is unclear why the loss of Dicer causes down-regulation of retrotransposons at E13.5 PGCs, these results suggested that in contrast to piRNA [21], [23], Dicer-mediated miRNA machinery or Dicer itself is dispensable for the repression of retrotransposons in PGC development and neonatal spermatogonia.

### Arrest of spermatogenesis at early stage in DicerCKO testis

**Figure 4:**
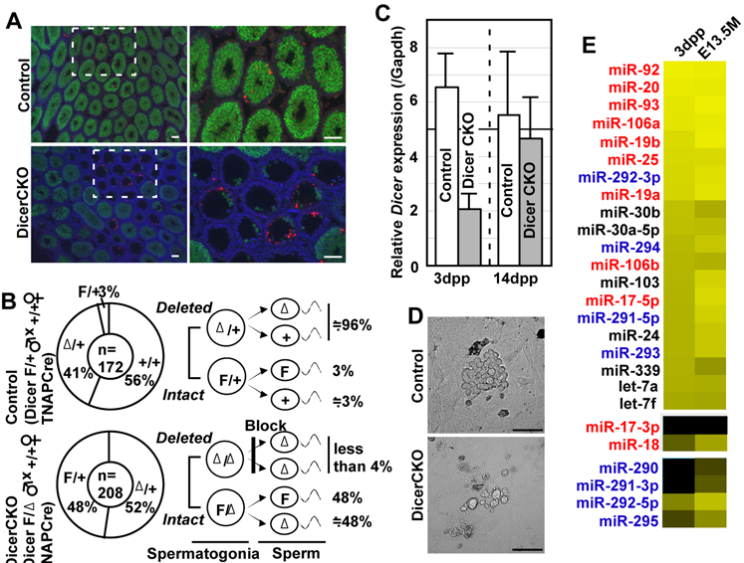
Crucial roles of Dicer on spermatogenesis. (A) Immunofluorescence analysis for Mvh (green), apoptotic cells (red) and nuclei (blue) in 4 weeks-old DicerCKO testis. Right images show high magnification of white broken rectangles in left images. Scale bar: 50 µm (B) Biased inheritance of floxed *dicer* allele by progeny from DicerCKO males. Pie charts summerise percentages of genotypes of progeny from Dicer^F/+^ TNAP-Cre (upper) and DicerCKO (bottom) males crossed with wild-type females. The number of progeny tested is shown in the center of the circles. Note that intact floxed *dicer* allele (F) is frequently inherited by progeny from DicerCKO males. Illustration in the right side of the pie charts estimates schematic ratio of *Dicer* excision in spermatogonia and spermatozoa, based on the percentages in the pie charts. Since floxed *dicer* allele is inherited by almost half of progeny from DicerCKO males, almost all fertile spermatozoa seems to be derived from Dicer^F/+^ spermatogonia which had escaped from *Dicer* excision, indicating blockage of spermatogenesis of *Dicer*-deleted cells. (C) Expression levels of *Dicer* in neonatal spermatogonia and 14 dpp spermatocytes. Relative expression levels of *Dicer* were calculated by reference to the levels of *Gapdh*. (D) Representative colonies derived from neonatal testis after culture for 2 weeks. Scale bar; 50 µm (E) The list of miRNA highly expressed in neonatal spermatogonia. MiRNAs encoded in miR-17-92 and miR-290-295 clusters are marked with red and blue, respectively. The miRNA levels detected by real-time PCR are represented as a heat map. The intensity of yellow scale corresponds to the level of miRNA expression; black indicates that the signal below detection levels (see in Figure 1A).

We next analysed spermatogenesis in DicerCKO testis. In 4 weeks-old DicerCKO testes, we frequently found seminiferous tubules that were entirely lacking in spermatogenesis (Figure 4A). In some 4 weeks-old DicerCKO testes, we also observed a low density of spermatogenic cells in the seminiferous tubes (Figure S6). A few spermatogenic cells, which were detected in the otherwise empty seminiferous tubules, showed expression of mouse vasa homolog (Mvh) that is normally expressed in spermatogonium to round spermatids stage during spermatogenesis [40]. Although apoptotic cells were also detected in 4weeks-old DicerCKO testis (Figure 4A), these were at a relatively low frequency compared to the massive apoptosis seen at the pachytene spermatocytes or round spermatids in piwi-deficient testes [20]–[22].

However, as shown in Figure 4A, some seminiferous tubules were full of spermatogenic cells in DicerCKO. In fact, 8 to 16 weeks-old DicerCKO males were fertile, though further older DicerCKO males (>8 month-old) were sterile because of eventually lacking spermatogenesis (Figure S6). We could not exclude the possibility that these spermatogenic cells were derived from spermatogonia that had escaped from the Cre-mediated *Dicer*-excision. To test for this possibility, DicerCKO males were mated with wild-type females to retrospectively determine whether an intact floxed *dicer* allele was present in some spermatogonia, which would then be inherited by the progeny. In control experiments, genotyping the progeny from crosses between Dicer^F/+^ TNAP-Cre males and wild-type females showed that intact floxed allele was seldom inherited by the subsequent generation (3%) (Figure 4B), demonstrating that around 94% of the floxed *dicer* alleles were deleted in Dicer^F/+^ TNAP-Cre males (Figure 4B). However, since almost 50% of the progeny from DicerCKO male inherited an intact floxed *dicer* allele (Figure 4B), spermatogenesis had been arrested in *Dicer*-deleted cells, which probably produce a few if any fertile spermatozoa (Figure 4B).

Additionally, we analysed the function of Ago2, which possesses catalytic activity for mRNA degradation, in spermatogenesis. In contrast to DicerCKO, mice lacking Ago2 in germ cells (Ago2CKO) generated by crossing *Ago2*-floxed mice [41] with TNAP-Cre mice, showed neither a morphological abnormality of the testis nor a bias in the genotype of the progeny (Figure S7). This demonstrates that the spermatogenic defect in DicerCKO is independent of Ago2.

Considering the defective proliferation of DicerCKO PGCs, the biased inheritance might be caused by poor proliferation of spermatogenic cells at an early stage. Supporting this idea, it was indicated that *Dicer*-deleted cells were disappearing during early spermatogenesis, since the expression levels of *Dicer* was restored in 14 dpp DicerCKO spermatocytes (Figure 4C). Furthermore, DicerCKO neonatal spermatogonia could only form small irregular colonies *in vitro* after culture for 2 weeks (Figure 4D), whereas multi-cellular colonies were often observed in the controls. MiRNAs belonging to miR-17-92 and miR-290-295 clusters were still highly expressed in neonatal spermatogonia and at almost the same levels as those in E13.5 PGCs (Figure 4E), although miR-290 and miR-291-3p were not detectable. Taken together, these results demonstrated that Dicer-mediated miRNA biogenesis plays a pivotal role in the proliferation of spermatogonia at early stages of spermatogenesis, and miR-17-92 cluster may be responsible for their proliferation.

## Discussion

### Characteristic pattern of miRNA expression in PGCs

Our study here revealed that miR-17-92 cluster miRNAs were highly expressed throughout development of PGCs. MiR-17-92 cluster has been thought to act as oncogene in mammalian cells since it is highly expressed in many types of tumors, although expression of most miRNAs is lower in cancer cells than in normal tissues [30], [42]–[46]. Overexpression of a subset of miR-17-92 cluster accelerated the development of the B cell lymphoma [30], which is implicated in enhanced cell cycle progression [47] and inhibition of apoptosis in cancer cells [43]. Furthermore, miR-17-5p, -18, -19a and -19b decreased only in female PGCs as they quit mitotic proliferation and commence an entry into meiosis. These results suggest that miR-17-92 cluster plays a pivotal role in cell cycle progression in PGCs. These possibilities would be confirmed by disruption of individual miRNAs.

Most importantly, one of the predicted targets of miR-19a and -19b is the tumor suppressor PTEN [48], which negatively controls proliferation of PGCs. The reduction of PTEN promotes proliferation of PGCs [49], [50]. MiR-19a and -19b may regulate PTEN dosage, and consequently regulate PGC proliferation. In fact, our functional analysis showed that the number of PGCs in DicerCKO embryos was decreased due to a defect of proliferation rate rather than apoptosis. These observations are also similar to those in *Dicer*-deficient ES cells that showed slow proliferation and a decrease in G2/M phase [6], [7].

In addition to miR-17-92 cluster, it is notable that miR-290-295 cluster was also highly expressed in PGCs throughout their development. These transcripts of the cluster were also observed preferentially in ES cells, but notably, they decreased in differentiating cells [51], suggesting the role of the cluster in pluripotency of ES cells. Taking into account the fact that PGCs have the potential to give rise to pluripotent embryonic germ cells, it is likely that the expression of the cluster miRNAs in PGCs may underlie the plasticity of PGCs to dedifferentiate into pluripotent EG cells. This speculation is supported by the evidence that EG cells could not established from E11.5 DicerCKO PGCs (unpublished data). It has been reported that the majority (about 40%) of all miRNAs are expressed from either miR-17-92 or miR-290-295 clusters in ES cells [52]. Our results estimated that in PGCs, 69% and 14% of miRNAs tested were miR-17-92 and miR-290-295 clusters, respectively. The similar expression patterns and their consequencess in *Dicer*-deficient PGCs and ES cells indicate that the regulatory network of miRNA is likely to be conserved in these cell types.

### Temporal regulation of miRNA expression in PGCs

Our analysis of miRNA expression between E9.5 and E13.5 PGCs showed that 4 miRNAs (miR-141, -200a, -200c and -323) were down-regulated during PGC development. Three of these miRNAs belong to miR-200 superfamily and share similar seed sequence. Bioinformatic analysis showed that miR-141 and -200c are encoded within 500 bp in the mouse genome, indicating that they are generated from the same pri-miRNA. Interestingly, the locus encoding miR-141/200c is close to the “pluripotent cluster” consisting of Nanog, Stella/PGC7/Dppa3 and Gdf3 on mouse chromosome 6 (∼2000 kb). Close association between miR-141/200c and the pluripotent cluster is also observed in the short arm of the human chromosome 12 (∼700 kb). In addition, there is a perfect match between the sequences of miR-141/200c in the mouse and human, suggesting that miR-141/200c may have similar functions in the two species. Notably, the genomic region including miR-141/200c and the pluripotent cluster is characterised as an oncogenic region for the formation of the germ cell tumor (GCT) in human [53], though the definitive connection with oncogenesis is yet unclear. Because it has been known that GCTs express stem cell-specific genes, including Nanog, which are also expressed in PGCs, it is possible that misregulation of miR-141/200c is a contributing factor for GCT formation. Supporting this speculation, a recent report showed that miR-141 functioned in promoting cell proliferation in cancer cell lines [54].

By contrast, the expressions of miRNAs belonging to let-7 family (let-7a, d, e, f and g) were up-regulated after E11.5 and reached the highest levels at E13.5. It is known that let-7 family functions in multiple differentiation events in various tissues and inhibit cell proliferation in cancer cells [34]. Interestingly, the up-regulation of the let-7 family was only observed in male PGCs. These results illustrate a possible mechanism involving miRNA regulating sex-specific PGC differentiation. In E13.5 male PGCs, it may be necessary to maintain high levels of miR-17-92 cluster for further proliferation of male germ cells after birth. Indeed, our results showed that neonatal spermatogonia also showed highly expressed miR-17-92 cluster and that DicerCKO neonatal spermatogonia showed poor proliferation. Perhaps, up-regulation of male-specific miRNA, including the let-7 family, may play a role in preventing cell cycle progression despite high levels of expression of miR-17-92 cluster.

### Dispensable function of miRNA in repression of transposable elements

One of the major roles of small RNAs is thought to maintain genome stability through suppression of transposable elements, based on widely known fact that mutants of RNAi machinery prevent silencing of transposable elements in many eukaryotes, including *Caenorhabditis elegans* [55], [56], *Drosophila melanogaster* [57], *Neurosporra crassa* [58] and *Trypanosma bruceii* [59], [60]. In the mouse, it has recently been reported that piRNAs play an essential role in repression of transposable elements [21], [23]. In addition to piRNA, since disruption of Dicer function lead to up-regulation of retrotransposon expression in preimplantation embryos and ES cells [6], [15], miRNAs have also been thought to play a role in the repression of retrotransposons. Our study, however, showed that LINE-1 and IAP expression were in fact down-regulated in DicerCKO PGCs and were comparable in DicerCKO neonatal spermatogonia, indicating at least that Dicer is dispensable for silencing these retrotransposons in the germ cell lineage. Previous reports and our results may illustrate the different functions of Dicer on repression of retrotransposons in PGCs and in preimplantation embryos or ES cells. An important difference amongst these cell types is that only PGCs and spermatogenic cells express piRNA-interacting proteins, Mili and Miwi2 [61]. Thus, the silencing retrotransposons may be governed by only piRNA in the germ cell lineage. Supporting this idea, in the *Drosophila* germline, Dicer-1 is not required for silencing retrotransposons, but instead, rasiRNAs of which function is associated with Piwi proteins is indeed required for their silencing [57]. Taken together, miRNAs and their biogenesis have a distinct pathway from piRNAs in silencing retrotransposons and in germ cell development. The reason why DicerCKO PGC showed subtle expression of the retrotransposons remains elusive. One plausible explanation is that miRNAs negatively regulate the expression of molecules that repress retrotransposons. Alternatively, loss of Dicer may enhance other small RNA pathway by conceding effecter molecules that are necessary for both small RNA pathways.

### Distinctive roles of miRNA in spermatogenesis

Although young (at least ∼16weeks old) DicerCKO males are fertile, genotyping these progeny revealed that the mature spermatozoa were derived from *Dicer* ^F/Δ^ spermatogonia, which had escaped from Cre-mediated *Dicer* excision. In the defective seminiferous tubules in 4weeks-old male, there were few meiocytes, indicating that spermatogenesis in DicerCKO was disturbed at an early stage before entering meiosis. Based on evidence that DicerCKO neonatal spermatogonia could seldom form a colony *in vitro*, it seems that the arrest of spermatogenesis is caused by the defect of proliferation and/or early differentiation of spermatogonia. According to aging, spermatogenic cells disappeared and then lacked in testes that were older than 8 month. To explain the reason, we set two possibilities that could not be evaluated in this study. One is that *Dicer* gene, which had escaped from Cre-mediated deletion until neonatal stage, was eventually excised during aging, resulting in loss of spermatogenesis in aged testis. The other is that ectopic deletion of *Dicer* gene in testicular somatic cells causes the defect of spermatogenesis.

Since miRNAs, Dicer and Ago2 are localized in the chromatoid body, which is a characteristic fibrous structure in the cytoplasm of spermatocyte and spermatid [26], it is possible that miRNAs play a role in late spermatogenesis. However, our results showed at least that Ago2CKO males were fertile and deleted *ago2* allele was inherited without bias in subsequent generation, demonstrating that Ago2 protein was dispensable for meiosis and spermiogenesis. Indeed, we observed characteristic spotty staining of Mvh outside the nuclei of almost all spermatids in Ago2CKO testis (Figure S7), indicating that the chromatoid body formed normally regardless of the absence of Ago2 protein. In this study, we could not see whether chromatoid body is formed in DicerCKO testis, because there are few *Dicer*-deleted meiocyte in the testis and it was difficult to clearly identify the few *Dicer*-deleted spermatids in DicerCKO testis that were present amongst many *Dicer*-undeleted cells. The function of Dicer in later spermatogenesis will be clarified by analysis using a different Cre transgenic mouse that is capable of deleting *Dicer* during or after meiosis. Nevertheless, taking together, all the observations and those from our study indicate that miRNAs are important for the proliferation and/or early differentiation of stem cell population in spermatogenesis.

## Materials and Methods

### Real-time PCR analysis of miRNAs

Real-time PCR analyses were performed according to a previous report [28]. Briefly, 10 PGCs were manually picked and put into a tube after PGCs were FACS-sorted from Oct-4ΔPE:GFP transgenic embryos. The PGCs were lysed and subjected to reverse transcription (RT) using specific primers for the 214 miRNAs. After the reaction, the RT product was used for Pre-PCR in which the cDNA of each miRNA can be representatively amplified by repeating 18 cycles of the reaction. The pre-PCR product was 1∶40 diluted and then used as a template DNA for real-time PCR using universal reverse primer and each miRNA-specific forward primers and TaqMan probes. To normalise values of the real-time PCR, recombinant miR-16 was used as a control. The raw values of each well were divided by the control value. Each miRNA reaction was done in duplicated wells. Three independent PGC samples at each developmental stage were used for the reaction. The relative values were converted into heat map using Cluster 3.0 and Java TreeView software.

### Genotyping of fetuses and mice

Genomic DNAs extracted from fetuses or mice were subjected to PCR analysis using primers: FN (GGT TAC ATG GCT AGA CTC AAA GC), RN (AGG TGC CTT TCG TTT AGG AAC) and FWF (AAA GCA GAA CTC TAA TGC CCC). PCR was performed in 25 µl reaction mixture containing REDTaq (Sigma) by the following heat cycles: 94°C for 30 s, 60°C for 30 s, 72°C for 60 s; 35 cycles.

### FACS analysis of the number of PGCs

Single cell suspensions were prepared from a pair of gonads by incubation with trypsin. The total number of cells in the suspension was counted. The percentage of GFP-positive cells was estimated by FACS, and then the number of PGCs was calculated by dividing the total number by the percentage.

### Immunostaining

To detect phosphorylated H3 Ser10, E12.5 gonads were fixed with 4% paraformaldehyde for overnight, washed with PBS and embedded in OCT Embedding Matrix (CellPath). The embedded gonad was sectioned, washed with PBS, and then incubated with primary antibodies; 1∶1000 diluted rabbit polyclonal anti-phospho-histone H3 Ser10 (Upstate) antibody and 1∶10 diluted mouse monoclonal anti-mouse ddx4 (Mvh) antibody. After reaction with secondary antibodies, the signals were detected by Radiance 2100 confocal microscopy (BioRad).

To detect Mvh protein and apoptotic cells, testes were isolated from individuals at each age, fixed with Bouin's fixative for 1 day, washed, dehydrated and embedded in paraffin. The embed testis was sectioned at 7 µm thick and then deparafined. After denaturing with boiled 10 mM sodium citrate buffer, the section was incubated with 1∶500 diluted rabbit polyclonal anti-mouse ddx4 (Abcam) followed by 1∶500 diluted goat polyclonal anti-rabbit-IgG conjugated with Alexa488 and DAPI. Apoptotic cells were detected using In Situ Cell Death Detection Kit TMR red (Roche).

### In situ hybridization and real-time PCR

In situ hybridization analysis was performed as previously described [62]. Probes for LINE1 were amplified from a genomic fragment that spanned the promoter region of a type A LINE1 element from nucleotides 1,211–1,628 in GenBank accession no. M13002. For real-time PCR, total RNA from FACS-sorted 3,000 PGCs was converted into cDNA using random primers. The cDNA was subjected to real-timePCR using SYBER green and specific primers; LINE1-F (TGG CTT GTG CTG TAA GAT CGA) and LINE-R (TTC CCA GTC TGT TGG TGG TCT) for LINE1 and IAP-F (TTT GAG GAG ACT GTA CCC CGA) and IAP-R (ATG ACG TTC AGC CGC AGT ATG) for IAP. Each value was divided by the value from *Gapdh* amplification using specific primer GAPDH-F (CAT GGC CTT CCG TGT TCC T) and GAPDH-R (GCG GCA CGT CAG ATC CA). As a negative control, the reaction mixture without reverse transcriptase was used.

For analysis of *Dicer* expression, cDNAs from sorted PGCs, neonatal spermatogonia or spermatocytes were subjected to real-time PCR. Neonatal spermatogonia were sorted from Oct4-GFP transgenic mice. Spermatocytes were fractionated by forward and side scatter. We confirmed that all of the sorted cells (105/105) were positive for Mvh by immunostaining. Real-time PCR analyses were performed using specific primers: Dicer/5143F (AAG ACT TGG AGT ACG GCT GCC T) and Dicer/5208R (TCT CTG CGT CTG GAT GGT CAA). Each value was divided by the value from *Gapdh* amplification.

### Culture of neonatal spermatogonia

Neonatal testis was dissociated and cultured as previously described [63]. Dissociated cells were cultured on gelatinised dish for 3 days. Then, the suspended cells were transferred onto mouse embryonic fibroblast treated with Mitomycin C and cultured for further 2 weeks. After culture, colonies composed of round cells were evaluated under microscope.

## Supporting Information

Figure S1 The list of 214 miRNAs tested. IDs of miRNAs and sequences are based on miRbase in Welicome Trust Sanger Institute (http://microrna.sanger.ac.uk/sequences/).
(1.10 MB EPS)

Figure S2 Profiling of miRNA expression during PGC development and in neonatal spermatogonia. The heat map represents all of miRNA expression levels tested. The intensity of yellow scale corresponds to the level of miRNA expression as described below the heat map. MiRNAs are listed, according to priority of high level of averaged miRNA expression during PGC development. Hierarchical tree shows similarities between each sample.
(0.92 MB TIF)

Figure S3 Efficiency of Cre-mediated Dicer excision in E13.5 PGCs. (A) PCR analysis using genomic DNAs from E13.5 sorted male PGCs and the tail. DNA fragments of Dicer gene locus were amplified by 28 cycles of PCR reaction (see materials and methods) and then loaded in an agarose gel. PCR products corresponding to each allele are indicated by arrowheads. Bars and the numbers below the gel image indicate the relative intensities between bands from F and deleted allele. (B) Expression levels of Dicer in E13.5 sorted male PGCs. Relative expression levels of Dicer were calculated by dividing with those of Gapdh. DicerF/+ TNAP-Cre fetuses were used as the controls.
(0.37 MB TIF)

Figure S4 Comparable level of apoptotic cells in DicerCKO PGCs at E13.5. Immunofluorescence analysis for Mvh (green), apoptotic cells (red) and nuclei (blue) in E13.5 DicerCKO and littermate control gonads. Two images are randomly chosen from each genotype. Scale bar; 50 µm
(1.54 MB TIF)

Figure S5 DNA methylation in LINE-1 and IAP sequences in E13.5 DicerCKO PGCs. Bisulfite sequencing profiles of LINE-1 and IAP sequences are shown with filled (methylated) and open (unmethylated) circles. Gaps in the methylation profiles represent mutated or missing CpG sites. The numbers under the bisulfite sequencing profiles show the percentages of methylated CpG.
(0.26 MB TIF)

Figure S6 Defective spermatogenesis in DicerCKO testes. (A) Hematoxylin and eosin (HE)-stained testicular sections from 4 weeks-old DicerCKO and littermate control males. Spermatogenic cells in DicerCKO testis are fewer than those in littermate control. (B) HE-stained (left) and immunostained (right) testicular sections from 8 month-old DicerCKO and littermate control males. Immunofluorescence analysis shows Mvh (green) and nuclei (blue).
(2.14 MB TIF)

Figure S7 Dispensable role of Ago2 in spermatogenesis. (A) Immunofluorescence analysis for Mvh (green) and nuclei (blue) in 4 weeks-old Ago2CKO testis. Middle image show high magnification of white broken rectangles in left image. Note that right image shows spotty staining of Mvh in spermatids. Scale bar: 50 µm (B) Genotypes of progeny and schematic ratio of genotype of spermatogonia and spermatozoa. The pie chart summerises percentages of genotypes of progeny from Ago2CKO males crossed with wild-type females. The number of progeny tested is shown in the center of the circle. Note that intact floxed ago2 allele (F) is inherited by only 7% of progeny from Ago2CKO males, similar to that from DicerF/+ TNAP-Cre males (Figure 4). Illustration in the right side of the pie chart estimates schematic ratio of Ago2 excision in spermatogonia and spermatozoa, based on the percentages in the pie chart. Since floxed ago2 allele is inherited by only 7% of progeny from Ago2CKO males, Ago2-deleted spermatogonia seems to develop normally into fertile spermatozoa.
(0.95 MB TIF)
