# Supplementary figures and images for: MicroRNA Biogenesis Is Required for Mouse Primordial Germ Cell Development and Spermatogenesis
Source: PLoS One. 2008 Mar 5;3(3):e1738. doi: 10.1371/journal.pone.0001738 (PMC2254191; doi:10.1371/journal.pone.0001738)

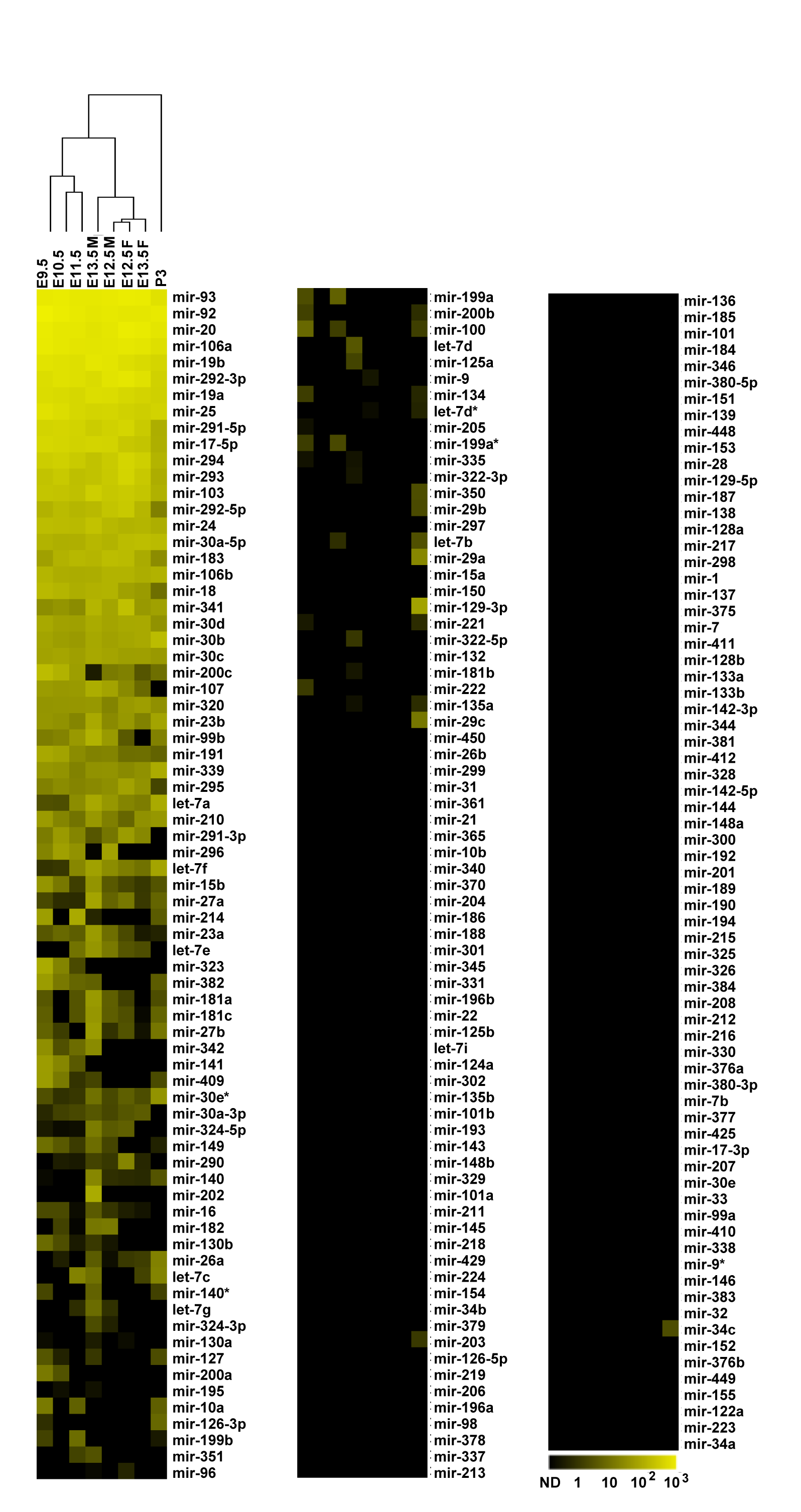

Supplement: Figure S2 — Profiling of miRNA expression during PGC development and in neonatal spermatogonia. The heat map represents all of miRNA expression levels tested. The intensity of yellow scale corresponds to the level of miRNA expression as described below the heat map. MiRNAs are listed, according to priority of high level of averaged miRNA expression during PGC development. Hierarchical tree shows similarities between each sample. (0.92 MB TIF) [file pone.0001738.s002.tif]

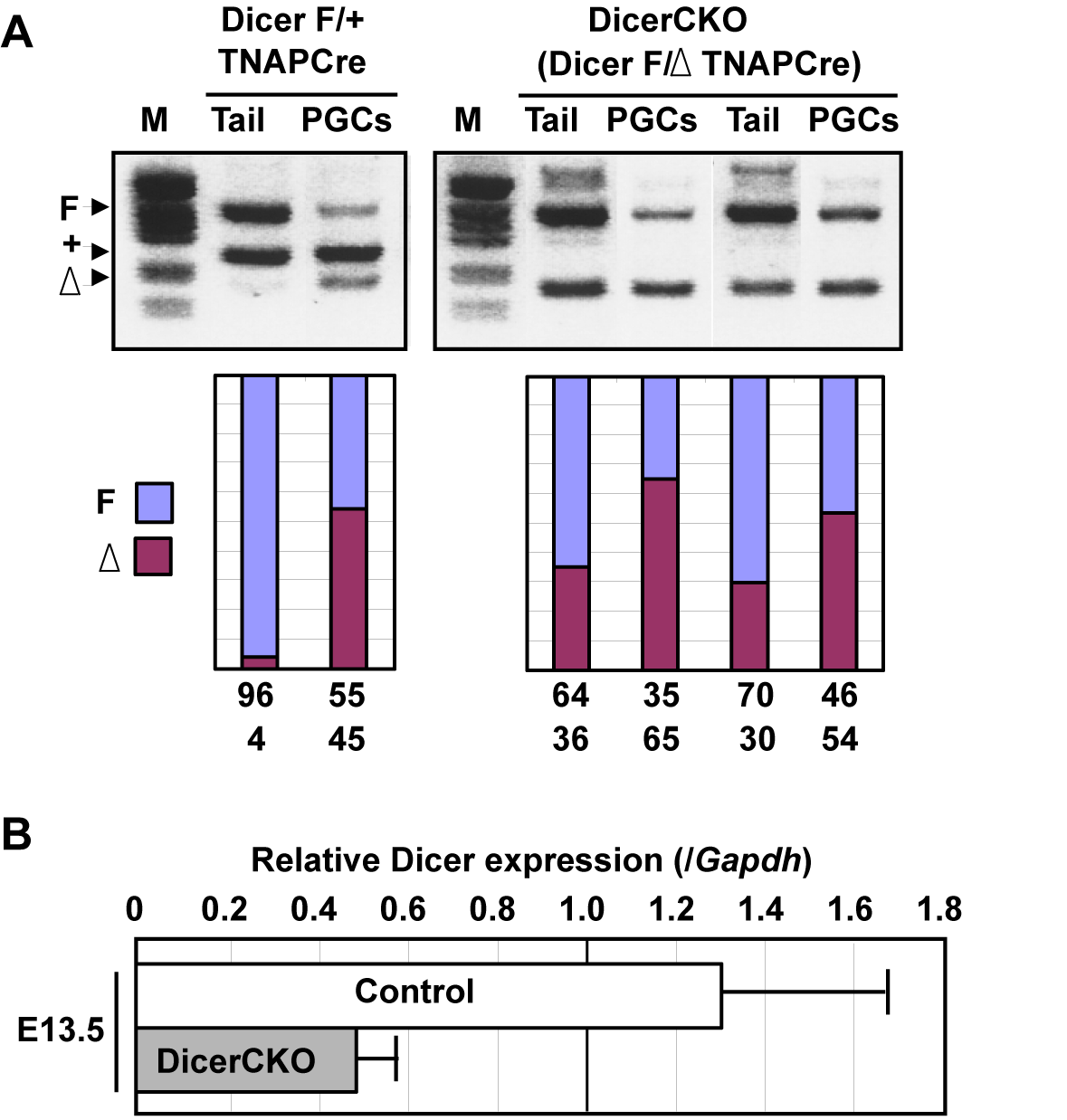

Supplement: Figure S3 — Efficiency of Cre-mediated Dicer excision in E13.5 PGCs. (A) PCR analysis using genomic DNAs from E13.5 sorted male PGCs and the tail. DNA fragments of Dicer gene locus were amplified by 28 cycles of PCR reaction (see materials and methods) and then loaded in an agarose gel. PCR products corresponding to each allele are indicated by arrowheads. Bars and the numbers below the gel image indicate the relative intensities between bands from F and deleted allele. (B) Expression levels of Dicer in E13.5 sorted male PGCs. Relative expression levels of Dicer were calculated by dividing with those of Gapdh. DicerF/+ TNAP-Cre fetuses were used as the controls. (0.37 MB TIF) [file pone.0001738.s003.tif]

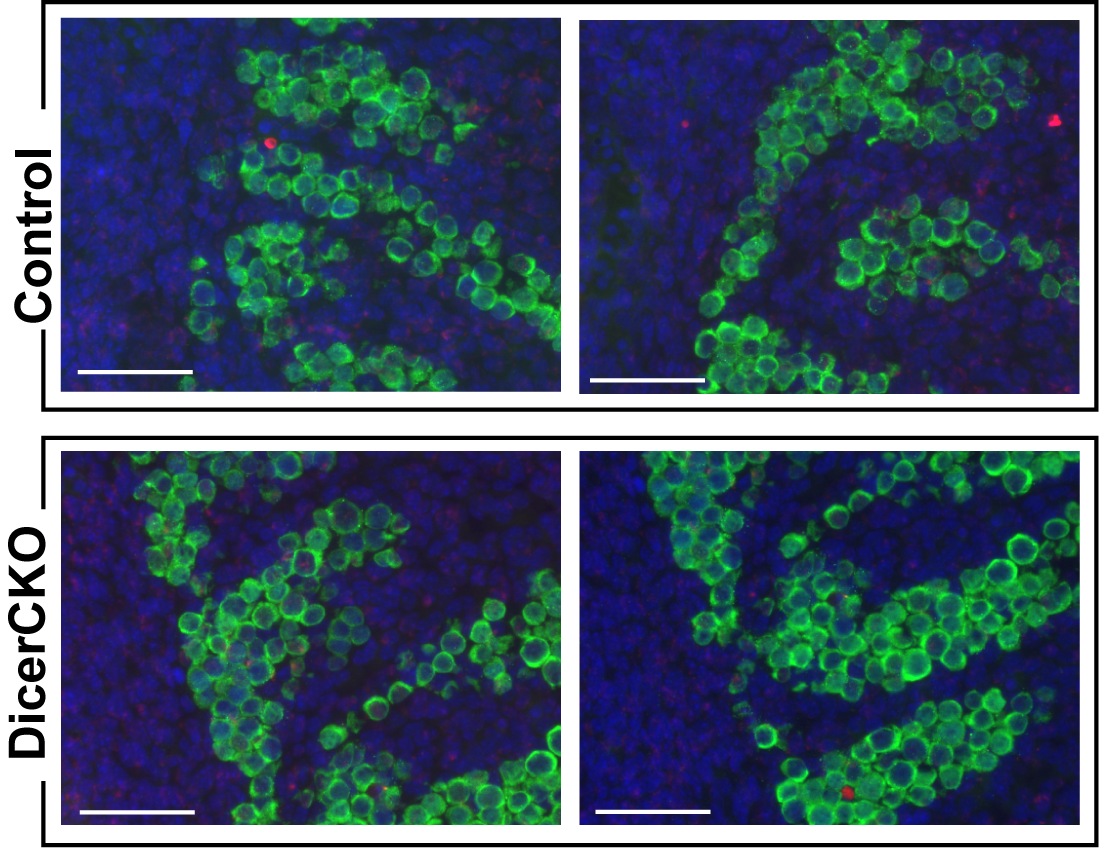

Supplement: Figure S4 — Comparable level of apoptotic cells in DicerCKO PGCs at E13.5. Immunofluorescence analysis for Mvh (green), apoptotic cells (red) and nuclei (blue) in E13.5 DicerCKO and littermate control gonads. Two images are randomly chosen from each genotype. Scale bar; 50 µm (1.54 MB TIF) [file pone.0001738.s004.tif]

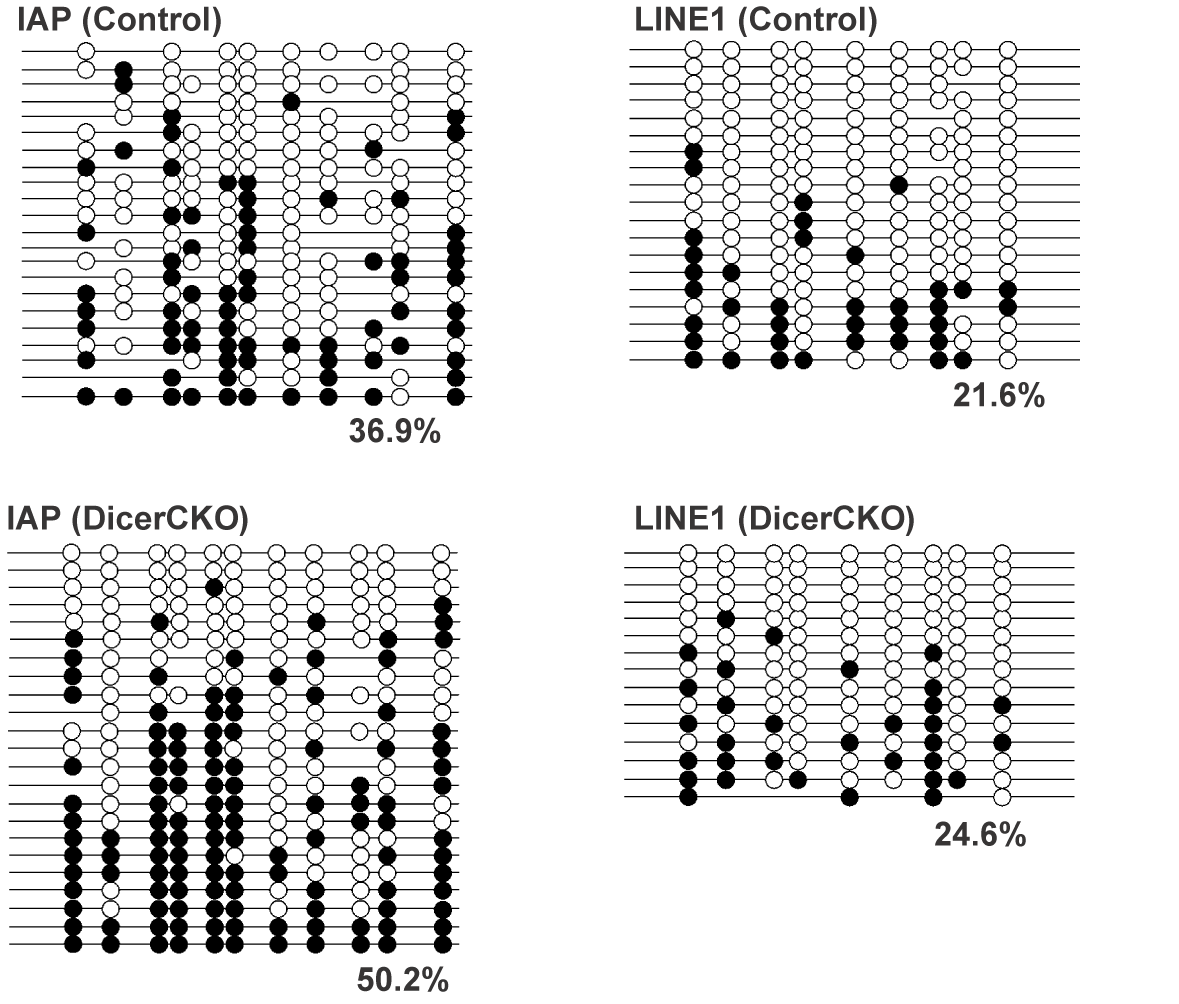

Supplement: Figure S5 — DNA methylation in LINE-1 and IAP sequences in E13.5 DicerCKO PGCs. Bisulfite sequencing profiles of LINE-1 and IAP sequences are shown with filled (methylated) and open (unmethylated) circles. Gaps in the methylation profiles represent mutated or missing CpG sites. The numbers under the bisulfite sequencing profiles show the percentages of methylated CpG. (0.26 MB TIF) [file pone.0001738.s005.tif]

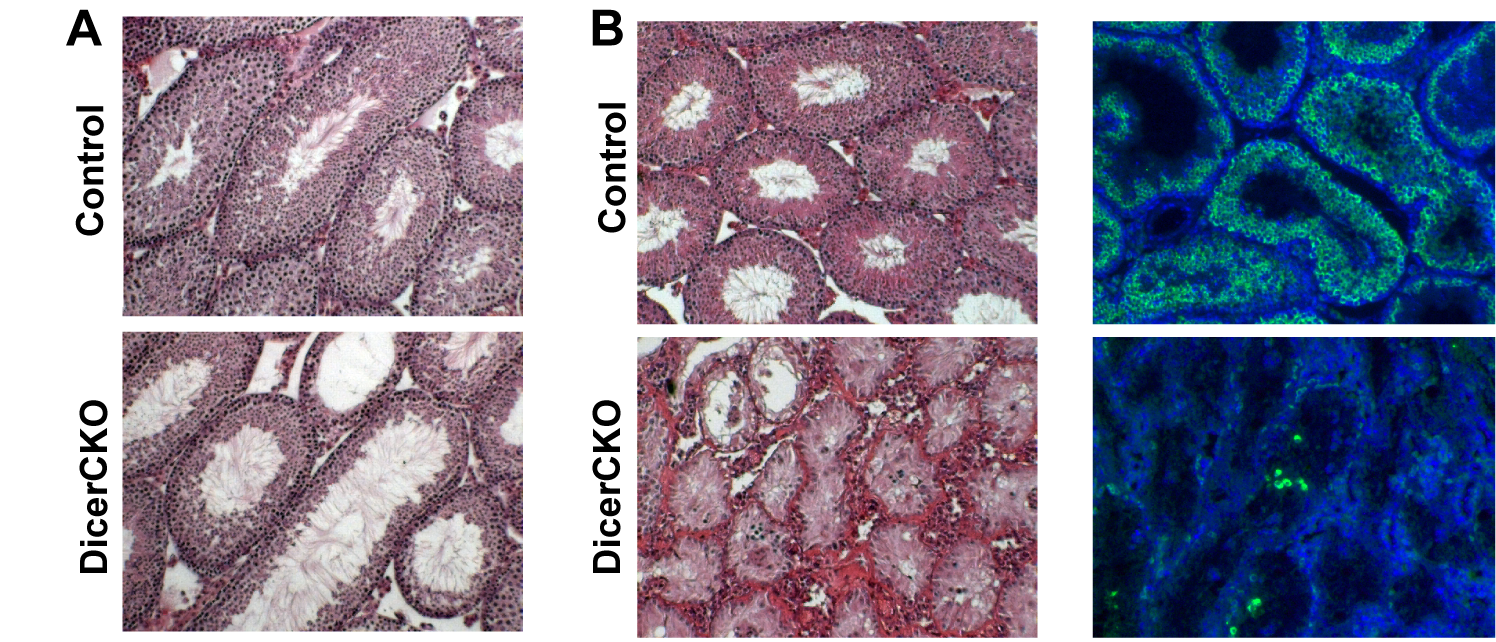

Supplement: Figure S6 — Defective spermatogenesis in DicerCKO testes. (A) Hematoxylin and eosin (HE)-stained testicular sections from 4 weeks-old DicerCKO and littermate control males. Spermatogenic cells in DicerCKO testis are fewer than those in littermate control. (B) HE-stained (left) and immunostained (right) testicular sections from 8 month-old DicerCKO and littermate control males. Immunofluorescence analysis shows Mvh (green) and nuclei (blue). (2.14 MB TIF) [file pone.0001738.s006.tif]

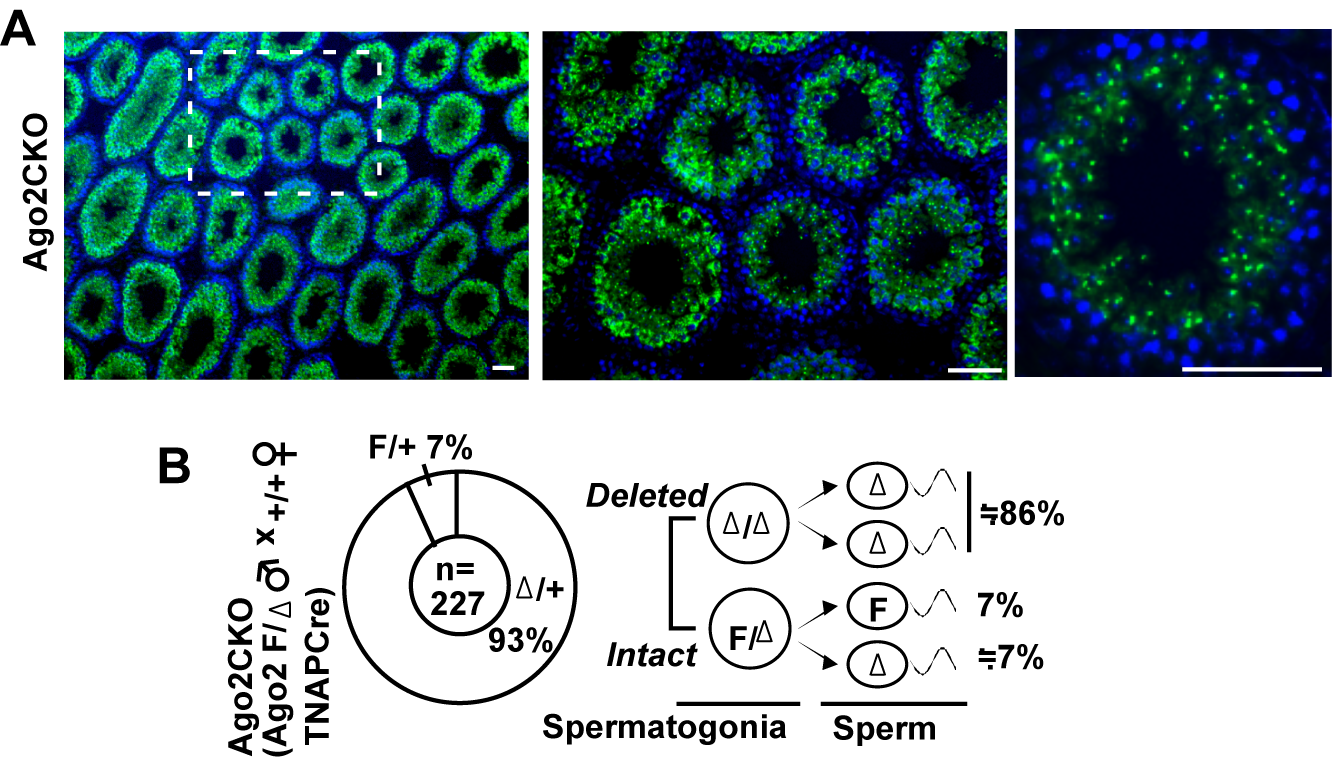

Supplement: Figure S7 — Dispensable role of Ago2 in spermatogenesis. (A) Immunofluorescence analysis for Mvh (green) and nuclei (blue) in 4 weeks-old Ago2CKO testis. Middle image show high magnification of white broken rectangles in left image. Note that right image shows spotty staining of Mvh in spermatids. Scale bar: 50 µm (B) Genotypes of progeny and schematic ratio of genotype of spermatogonia and spermatozoa. The pie chart summerises percentages of genotypes of progeny from Ago2CKO males crossed with wild-type females. The number of progeny tested is shown in the center of the circle. Note that intact floxed ago2 allele (F) is inherited by only 7% of progeny from Ago2CKO males, similar to that from DicerF/+ TNAP-Cre males (Figure 4). Illustration in the right side of the pie chart estimates schematic ratio of Ago2 excision in spermatogonia and spermatozoa, based on the percentages in the pie chart. Since floxed ago2 allele is inherited by only 7% of progeny from Ago2CKO males, Ago2-deleted spermatogonia seems to develop normally into fertile spermatozoa. (0.95 MB TIF) [file pone.0001738.s007.tif]
